# Huaier Suppresses Breast Cancer Progression via linc00339/miR-4656/CSNK2B Signaling Pathway

**DOI:** 10.3389/fonc.2019.01195

**Published:** 2019-11-08

**Authors:** Wei Wang, Xiaolong Wang, Chen Li, Tong Chen, Ning Zhang, Yiran Liang, Yaming Li, Hanwen Zhang, Ying Liu, Xiaojin Song, Wenjing Zhao, Bing Chen, Lijuan Wang, Qifeng Yang

**Affiliations:** ^1^Department of Breast Surgery, Qilu Hospital, Shandong University, Jinan, China; ^2^Department of Thyroid and Breast Surgery, Jining No. 1 People's Hospital, Jining, China; ^3^Department of Pathology Tissue Bank, Qilu Hospital, Shandong University, Jinan, China

**Keywords:** Huaier, breast cancer, bioinformatics, long non-coding RNA, microarray

## Abstract

Huaier, as known as *Trametes robiniophila* Murr, is a traditional Chinese medicine. Various studies have demonstrated that Huaier could inhibit cancer progression and improve the prognosis of patients. In the present study, we comprehensively screened the expression profiles of lncRNAs, miRNAs, and mRNAs in Huaier-treated breast cancer cells. Using bioinformatic analysis, hub genes were identified and functionally annotated. Weighted gene coexpression network analysis was applied to construct the molecular network influenced by Huaier. Linc00339 was then found to play a critical role in Huaier-mediated cancer suppression. To validate the effects of linc00339 and identify the downstream targets, we performed *in vitro* and *in vivo* experiments. Finally, we identified that Huaier could inhibit the proliferation of breast cancer cells through modulating linc00339/miR-4656/CSNK2B signaling pathway.

## Introduction

Huaier, also known as *Trametes robiniophila Murr*, is a sandy beige mushroom. It has been applied as a traditional Chinese medicine (TCM) for more than 1,600 years. Huaier aqueous extract and Huaier granule, whose commercial name is Jinke, are the most common types used in clinical treatments. According to the HPLC (high performance liquid chromatography) and SDS-PAGE (polyacrylamide gel electrophoresis) analysis, the most effective ingredient of Huaier is identified as proteoglycan, which includes 41.53% polysaccharides, 12.93% amino acids and 8.72% water (1). Polysaccharide was identified as a possible key ingredient in Huaier (2–4). Previous studies showed that Huaier exerted therapeutic effects on psoriasis (5), inflammation (6), and tuberous sclerosis (7). In dextran sulfate sodium-induced experimental colitis, Huaier suppressed the secretion of interleukin 1β (IL-1β) and the activation of caspase-1 via inhibiting NLRP3 inflammasome (8). In the anti-Thy-1 mesangial proliferative glomerulonephritis (MsPGN), Huaier could inhibit the urinary protein excretion and reduce hyperplasia (9).

Increasing evidence demonstrated that Huaier presents potent anti-neoplastic activities in various tumors, such as colon cancer (10), fibrosarcoma (11), and cervical cancer (12). In a multicenter, randomized clinical trial, Huaier granule could significantly improve the recurrence-free survival and reduce extrahepatic recurrence in hepatocellular carcinoma (HCC) patients who received radical surgical resection (13). In lung cancer, Huaier suppressed the proliferation and metastasis of cancer cells through inducing apoptosis and cell cycle arrest. MTDH, JAK2/STATS, and MAPK signaling pathways were involved in the inhibitory effects of Huaier (14). Additionally, Huaier could regulate the activation of PI3K/AKT signaling pathway, and modulate the expression of cyclin B1 in gastric cancer cells (15).

Our previous study demonstrated that Huaier granule could improve the disease-free survival of breast cancer patients from 91.43 to 112.61 months with higher KPS scores and less emotional symptoms (16). To further determine the molecular mechanisms, we screened the expression profiles of triple-negative breast cancer cells after Huaier treatment, and multi-target effects of Huaier have been identified (17). We also found that Huaier aqueous extract could induce apoptosis and cause cell cycle arrest at G0/G1 phase (1). In which the death-receptor pathway and the mitochondrial pathway contributed to the anti-cancer effects of Huaier (1). In the ER-positive breast cancer cells, Huaier significantly disturbed the estrogen receptor α signaling pathway (18) and inhibited the self-renewal activity of breast cancer stem cells through inactivation of hedgehog pathway (19). Furthermore, Huaier could inhibit angiogenesis without obvious toxicity to mice *in vivo* (20) and induce autophagy via suppressing mTOR/S6K pathway (21).

Non-coding RNAs played critical roles in the cancer progression. According to our data, lncRNA-H19/miR-675-5P/CBL axis was involved in the inhibitory effects of Huaier extract (22). However, the competing endogenous RNA (ceRNA) network caused by Huaier in breast cancer cells has not been studied. Here, we analyzed the expression profiles of lncRNAs, miRNAs, and mRNAs in Huaier-treated breast cancer cells. Through bioinformatic analysis, linc00339 was identified as the hub gene in the function of Huaier extract. Using public databases, the clinical significances of linc00339 and its downstream targets were discovered. Finally, *in vitro* and *in vivo* experiments confirmed that linc00339/miR-4656/CSNK2B signaling pathway played a critical role in the anti-cancer effects of Huaier extract.

## Materials and Methods

### Cell Lines and Reagents

The human breast cancer cell lines MDA-MB-231 and MCF7 were purchased from American Type Culture Collection (ATCC, Manassas, VA, USA), and routinely maintained in DMEM/high glucose medium (Gibco-BRL, Rockville, IN, USA) supplemented with 10% fetal bovine serum (Haoyang Biological Manufacture, Tianjin, China), 100 U/ml penicillin and 100 μg/ml streptomycin in 5% CO2 at 37°C. Huaier was kindly provided by Gaitianli Medicine Co., Ltd. (Jiangsu, China) and was prepared as previously described (20). MicroRNA mimics and siRNAs were obtained from GenePharma (Shanghai, China). In order to inhibit linc00339, the following siRNA sequences were targeted: linc00339 siRNA 1: 5′-GCCAGAAGUUGUCCUACUATT-3′; linc00339 siRNA 2: 5′-GGGAUGUCCUCAGGCAUCTTT-3′.

### Profiling of lncRNA, miRNA, and mRNA Expression

Affymetrix GeneChip Human Transcriptome Array 2.0 was used to profile the expression of lncRNAs, miRNAs, and mRNAs, which was performed by GMINIX BioTech (Shanghai, China). Sample labeling, microarray hybridization and washing were performed according to the manufacturer's standard protocol (23). In brief, RNA samples from breast cancer cells (MDA-MB-231 and MCF7) with or without Huaier treatment were extracted using TRIzol Reagent and synthesized to biotinylated cDNA. Then cDNA was synthesized and hybridized to the microarray. After hybridization and washing, the arrays were scanned by Affymetrix Microarray Scanner. Raw data of Affymetrix GeneChip Human Transcriptome Array 2.0 were extracted and normalized by Affymetrix Transcriptome Analysis Console Software (Version 4.0, Affymetrix). Expression Console (Version 1.3.1, Affymetrix) software performed RMA (Robust Multichip Analysis) normalization for gene analysis. Fold change was used to identify deregulated genes.

### Identification of Differentially Expressed Genes

R package “limma” was applied to identify the differentially expressed genes (24). We searched for the differentially expressed genes using the following criteria. Fold change > 1.5 (criteria for mRNA) or > 1.3 (criteria for lncRNA and miRNA) was applied to find the upregulated and downregulated genes. *P*-value <0.05 were considered as significant changes. The corresponding heatmaps were drawn by R package “pheatmap” (25).

### GO and KEGG Analyses

Gene Ontology (GO) analysis was performed to annotate the biological importance of the differentially expressed genes, including cellular component, biological process, and molecular function. KEGG (Kyoto Encyclopedia of Genes and Genomes) pathway analysis was used to identify crucial pathways based on the deregulated genes. The important GO and KEGG terms were identified by Fisher's exact test, and FDR was applied to correct the *P*-values.

### Construction of the Coexpression Molecular Network

The construction of the coexpression molecular network was based on the weighted correlation network analysis (WGCNA), a comprehensive collection of R functions for performing various aspects of weighted correlation network analysis (26). The functions of WGCNA included network construction, module detection, gene selection, calculations of topological properties, data simulation, and visualization. Through using soft threshold, WGCNA could provide more extensive edges between different transcripts. The power of soft threshold was set to 18. And the genes in Module yellow (0.74) were selected. Nodes and edges in Module yellow were imported into Cytoscape, and the coexpression molecular network was finally built.

### Gene Set Enrichment Analysis (GSEA)

To evaluate the effect of linc00339 on different biological function gene sets in breast cancer cells, the mRNAs coexpressed with linc00339 were analyzed by GSEA. The reference gene sets were based on the Molecular Signatures Database (MSigDB), including H (hallmark gene sets), C1 (positional gene sets), C2 (curated gene sets), C3 (motif gene sets), C4 (computational gene sets), C5 (GO gene sets), C6 (oncogenic signatures), and C7 (immunologic signatures). The number of permutations was set at 1000. *P* < 0.05 and FDR < 0.25 were considered significant difference.

### Quantitative Real-Time PCR (qPCR) Analysis

Total RNA was isolated from breast cancer cells using TRIzol reagent (Invitrogen, Carlsbad, CA, USA) according to the manufacturer' instructions. mRNAs were reverse-transcribed into cDNAs using PrimeScript reverse transcriptase reagent kit (TaKaRa, Shiga, Japan). miRNAs were synthesized through using the Mir-XTM miRNA first-strand Synthesis Kit (TaKaRa, Shiga, Japan). The specific primers were as follows: linc00339 forward 5′-TTTGTGGGAGTTAGGGTCTTATC-3′, linc00339 reverse 5′-CTCGTGGAATCTGGACCTGG-3′.

### MTT (3-(4,5-dimethyl-2-thiazolyl)-2,5–diphenyl-2H-tetrazolium Bromide) Assay

Cell proliferation assay was performed using MTT (Sigma, St. Louis, MO, USA) according to the instructions. Cells were plated into 96-well cell culture plates with at least three replicate wells for each group. After treatment for indicated time, 20 μl of MTT was added into each well and incubated for another 4–6 h. Absorbance values were measured by a Microplate Reader (Bio-Rad) at 490 and 570 nm.

### Dual Luciferase Assay

The regions of Linc003339 or 3′UTR locus of CSNK2B, containing miR-4656-binding sites was amplified and cloned into pmirGLO luciferase miRNA Target Expression vector (Promega, Madison, WI, USA). The mutant reporter plasmids were constructed as described previously (27). The wide type plasmids (WT) or mutant type plasmids (Mut) were co-transfected with miR-4656 or NC into breast cancer cells. Firefly and Renilla luciferase activities were measured with the dual-luciferase reporter assay kit (Promega, Madison, WI, USA) 2 days after transfection. The relative luciferase activity was measured as the ratio of firefly luciferase activity to renilla luciferase activity. All experiments were performed in triplicate.

### *In vivo* Xenograft Tumorigenicity Assay

The animal experiments were performed in strict accordance with the Guidelines for the Care and Use of Laboratory Animals of Shandong University. The study was approved by the Ethics Committee on Scientific Research of Shandong University, Qilu Hospital. MDA-MB-231 cells transfected with empty vector or Linc00339 were injected subcutaneously into the right flank of 4–5 weeks old BALB/c nu/nu female mice. After 2 days, the mice were randomly divided into vehicle group and Huaier group. The Huaier group was given a 100 μL solution containing 50 mg Huaier, while the vehicle group was given 100 μL water. Drugs were administered by gavage daily. The volumes of tumors were recorded every 4 days. And the tumor volume was calculated as: *volume* = *width*^2^ × *length* ÷ *2*. After 26 days, the mice were sacrificed and the xenografts were removed for further study.

### Statistical Analysis

The software SPSS (version 18.0) was used for statistical analysis. A student's *t*-test and one-way ANOVA were performed to determine significance. All error bars represented the standard errors of three experiments and differences with *P* < 0.05 were considered significant.

## Results

### Screening of Differentially Expressed lncRNAs, miRNAs, and mRNAs in Huaier-Treated Breast Cancer Cells

**Figure 1:**
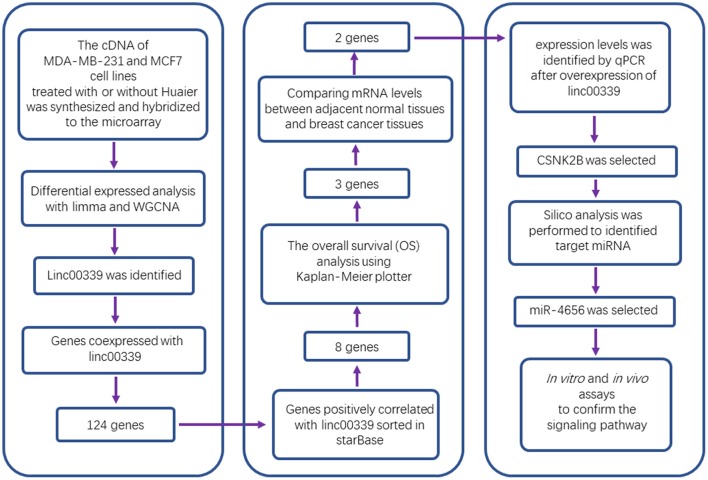
Study flowchart.

**Figure 2:**
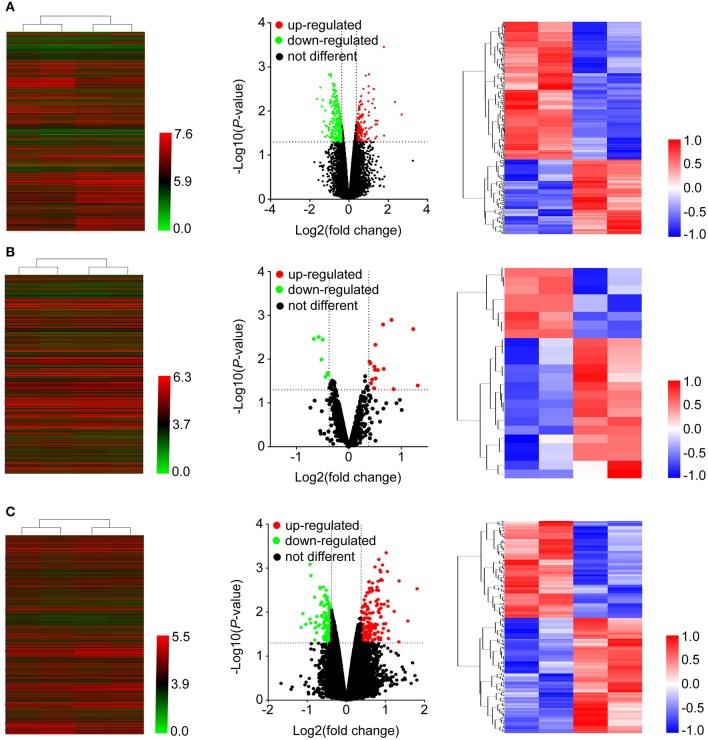
Heatmap and volcano plots were used to show the expression profiles of mRNAs **(A)**, miRNAs **(B)**, and lncRNAs **(C)**. Left panels, expression values of all the transcripts. Middle panels, differentially expressed transcripts and unchanged transcripts labeled by different colors. Right panels, differentially expressed transcripts shown in the heatmap.

As shown in Figure 1, after treatment with 8mg/ml Huaier for 72h, breast cancer cells were collected and analyzed with Affymetric GeneChip Human Transcriptome Array 2.0. Heatmaps were used to show the expression levels of all the probes. In total, 26695 mRNAs (Figure 2A), 1243 miRNAs (Figure 2B), and 39610 lncRNAs (Figure 2C) were detected. To investigate the differentially expressed genes, we set the thresholds as fold change ≥ 1.3-fold and *P* < 0.05. Volcano plots were used to show the deregulated genes after Huaier treatment. Among them, 487 mRNAs had differential expressions in Huaier-treated breast cancer cells, consisting of 316 downregulated mRNAs and 171 upregulated mRNAs. Of the 24 deregulated miRNAs, 8 were downregulated and 16 were upregulated. Of the 313 deregulated lncRNAs, 143 lncRNAs were upregulated and 170 lncRNAs were downregulated. Hierarchical clustering analysis presented systematic variations between Huaier-treated group and control group. These results suggested that Huaier induced significant changes of mRNA, miRNA, and lncRNA levels in breast cancer cells.

### Functional Analysis of Differentially Expressed Genes

**Figure 3:**
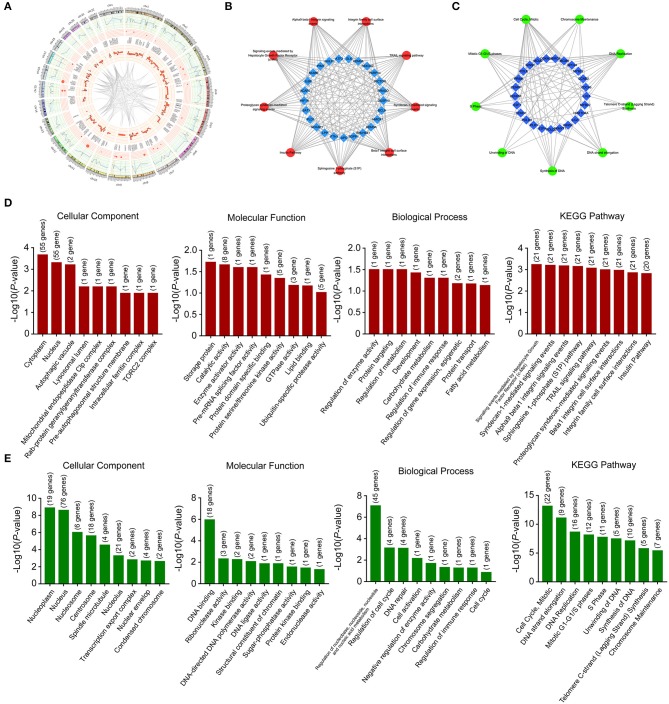
Location and functional annotation of differentially expressed transcripts. **(A)** Circos plot showing deregulated mRNAs, miRNAs and lncRNAs on human chromosome. **(B)** Interaction between significant pathways and upregulated mRNAs. **(C)** Interaction between significant pathways and downregulated mRNAs. **(D)** GO and KEGG pathway annotations of upregulated mRNAs. **(E)** GO and KEGG pathway annotations of downregulated mRNAs.

These deregulated mRNAs, miRNAs, and lncRNAs were widely distributed in all chromosomes (Figure 3A). Through analyzing differentially expressed mRNAs, we could indicate the roles of Huaier in breast cancer cells. The GO (gene ontology) and KEGG (Kyoto Encyclopedia of Genes and Genomes) pathway analyses could show the molecular processes after Huaier treatment. We first used the upregulated and downregulated mRNAs for biological pathway analysis. The pathway networks were constructed using the most enriched pathways to illustrate the critical genes in the process of Huaier treated breast cancer cells (Figures 3B,C). As shown in Figure 3D, the most enriched terms of cellular component mediated by upregulated mRNAs were cytoplasm, nucleus, and autophagic vacuole. In terms of molecular function, upregulated mRNAs were mostly enriched in storage protein, catalytic activity, and enzyme activator activity. In terms of biological process, the upregulated mRNAs were mainly associated with the regulation of enzyme activity, protein targeting and regulation of metabolism. In addition, the upregulated mRNAs were enriched in three KEGG pathways, which contained signaling events mediated by hepatocyte growth factor receptor (c-Met), syndecan-1-mediated signaling events and alpha9 beta1 integrin signaling events.

Through analyzing downregulated mRNAs (Figure 3E), the most enriched terms of cellular component were nucleoplasm, nucleus, and nucleosome. In terms of molecular function, downregulated mRNAs were mostly enriched in DNA binding, ribonuclease activity and kinase binding. In terms of biological process, downregulated mRNAs were strongly associated with regulation of nucleobase, nucleoside, nucleotide and nucleic acid metabolism, regulation of cell cycle and DNA repair. The most enriched KEGG pathway of downregulated mRNAs were cell cycle, mitotic, DNA strand elongation, and DNA replication.

**Figure 4:**
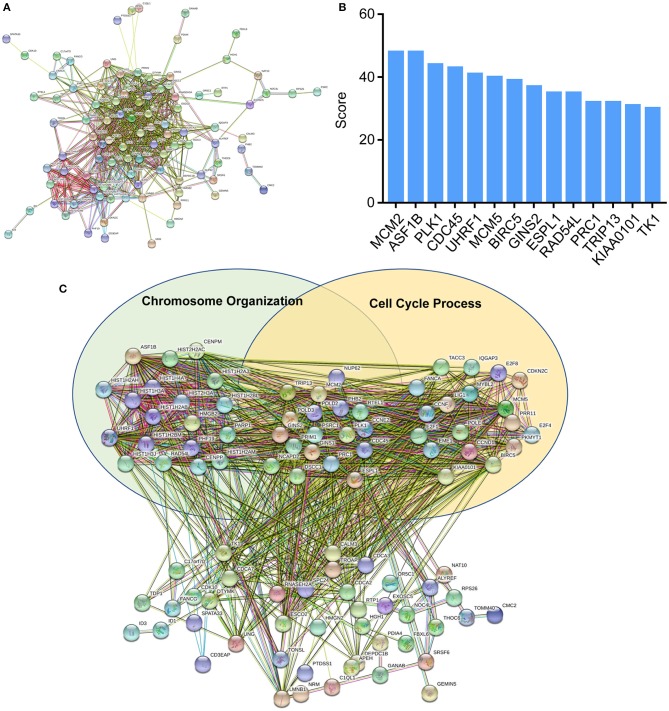
Protein-protein interaction (PPI) networks construction. **(A)** Interactions between differentially expressed mRNAs were analyzed by STRING. **(B)** Top *k*-score genes were shown in the histogram. **(C)** Deregulated mRNAs were involved in the two GO items, chromosome organization and cell cycle process.

To further explore the functions of deregulated mRNAs and identify hub genes, we used STRING for the construction of biological network and visual exploration (Figure 4A). A biological software, cytoscape, was adopted to calculate the k-score and assess the importance of genes (28). The 14 highest k-score genes were identified (Figure 4B). Using GO analysis, the networks were enriched in the DNA replication and cell cycle (Figure 4C).

### LncRNA/miRNA/mRNA Coexpression Network in Huaier-Treated Breast Cancer Cells

**Figure 5:**
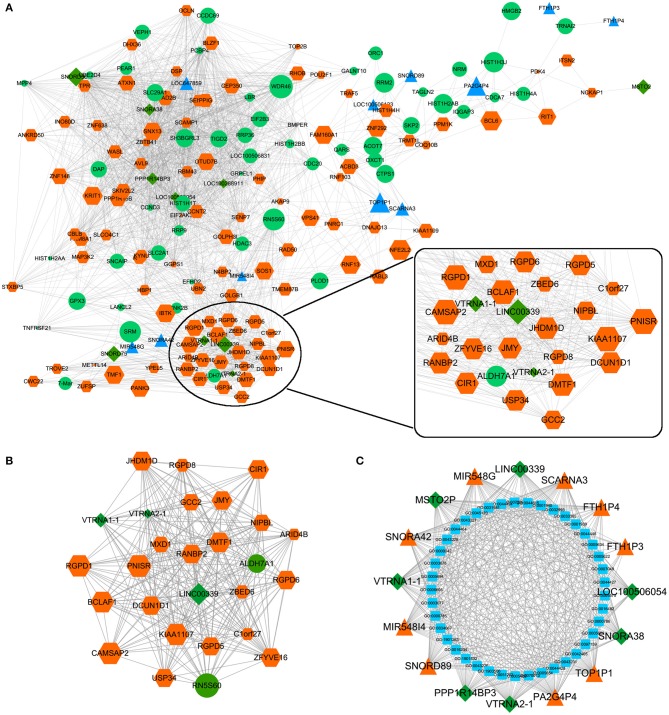
Coexpression network was constructed by WGCNA. **(A)** The whole coexpression network. Different sizes and colors represented the corresponding RNA types and expression levels. **(B)** The highest *k*-score subnetwork was identified. **(C)** Functional annotation of the subnetwork.

The effects of Huaier involved multiple transcriptomes and the interactions between transcriptomes formed a huge network. We used weighted gene coexpression network analysis (WGCNA), an R package for weighted correlation network analysis, to identify candidate therapeutic targets based on RNA sequencing (26). As shown in Figure 5A, the coexpression network contained 19884 edges and 208 nodes. In addition, several prominent subnetworks were constructed. The most stable subnetwork was formed by the hub transcripts regulated by Huaier treatment. The crucial subnetwork comprised of several lncRNAs and mRNAs (Figure 5B), in which linc00339 was located in the core of the subnetwork. Through GO analysis, the functions of the subnetwork were predicted (Figure 5C).

### Prediction of the Targets and Functions of linc00339

**Figure 6:**
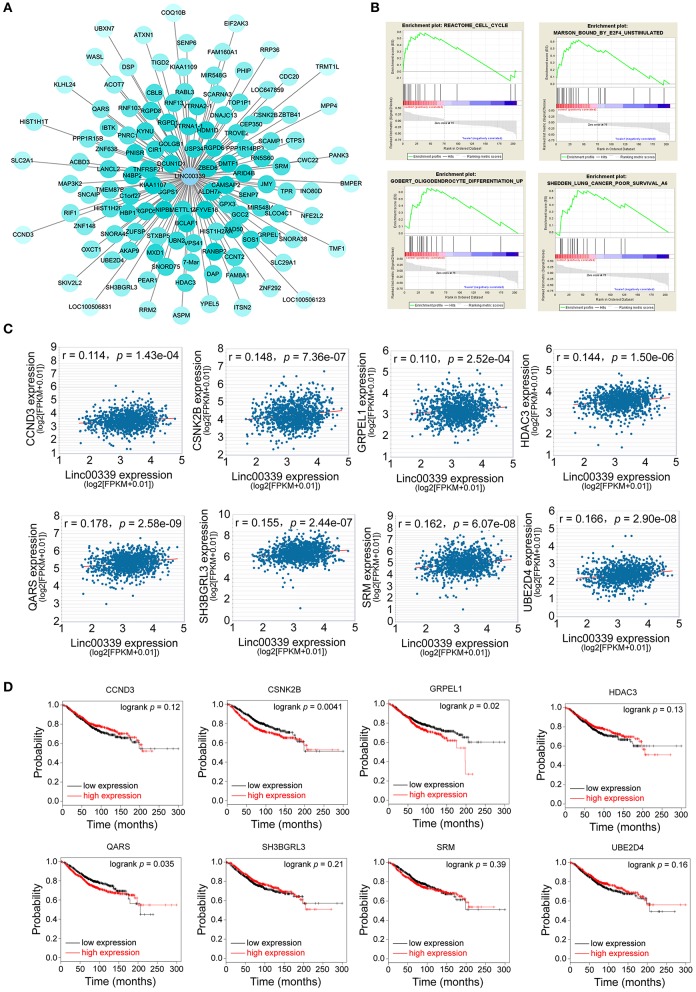
Exploration of the functions and associated genes of linc00339. **(A)** Transcripts coexpressed with linc00339. Colors were based on the Pearson's correlation. **(B)** GSEA for the mRNAs coexpressed with linc00339. **(C)** Pearson's correlation of linc00339 with 8 mRNAs. **(D)** Kaplan-Meier survival curves of 8 mRNAs predicted the overall survival of breast cancer patients in the TCGA database.

lncRNA could regulate the expression of miRNA and mRNA through multiple mechanisms (29–31). The miRNAs and mRNAs coexpressed with linc00339 were shown in Figure 6A, and the distances were based on the weights. Then the mRNAs coexpressed with linc00339 were annotated by gene set enrichment analysis (GSEA). The most enriched GSEA terms were reactome cell cycle, marson bound by E2F4 unstimulated, gobert oligodendrocyte differentiation up and shedden lung cancer poor survival A6 (Figure 6B).

Growing evidence indicated that lncRNAs could function as ceRNAs through sponging specific miRNAs and inhibiting the translation of downstream mRNAs (32). To further identify the downstream targets of linc00339, we used starBase to calculate the Spearman's correlation of linc00339 with its coexpressed mRNAs (33). As shown in Figure 6C, eight mRNAs were identified to be positively correlated with linc00339, including CCND3, CSNK2B, GRPEL1, HDAC3, QARS, SH3GRL3, SRM, and UBE2D4. The overall survival (OS) was analyzed using Kaplan-Meier plotter. According to the median value of candidate mRNAs expression, 3951 breast cancer patients were divided into high expression and low expression groups for each mRNA. As shown in Figure 6D, high expression of CSNK2B (*p* = 0.0041, HR = 1.37), GRPEL1 (*p* = 0.0200, HR = 1.29) and QARS (*p* = 0.0350, HR = 1.26) were corelated with worse prognosis in breast cancer patients, whereas CCND3, HDAC3, SH3BGRL3, SRM, and UBE2D4 were not correlated with OS.

**Figure 7:**
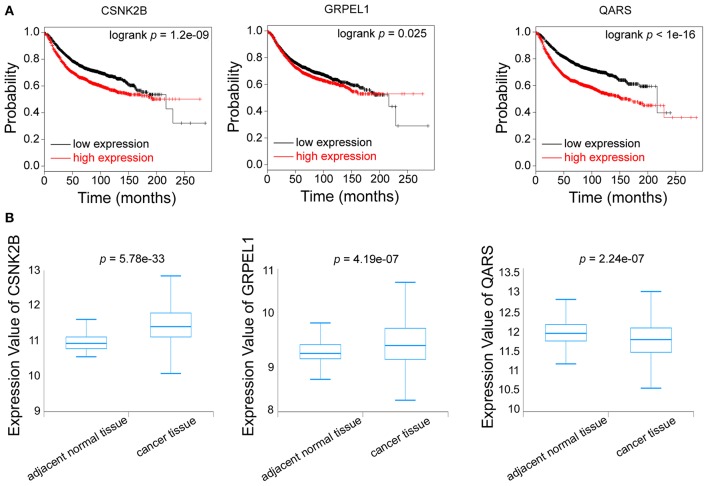
Identification of the downstream targets of linc00339. **(A)** Kaplan-Meier survival curves of CSNK2B, GRPEL1, and QARS predicted the disease free survival of breast cancer patients in the TCGA database. **(B)** Expressions of CSNK2B, GRPEL1, and QARS in samples from adjacent normal tissues and breast cancer tissues.

Furthermore, we evaluated the influences of CSNK2B, GRPEL1, and QARS on recurrence free survival (RFS). High expressions of all the three genes could lead to shorter RFS time (Figure 7A). Through comparison of mRNA levels between adjacent normal tissues and breast cancer tissues (Figure 7B), we found that CSNK2B (*p* = 5.78e-33) and GRPEL1 (*p* = 4.19e-07) were significantly upregulated in tumor tissues. However, higher level of QARS was detected in adjacent normal tissues (*p* = 2.24e-07). Therefore, CSNK2B and GRPEL1 could be the potential downstream targets of linc00339.

### Validation of the Molecular Mechanism Underlying the Inhibitory Effects of Huaier

**Figure 8:**
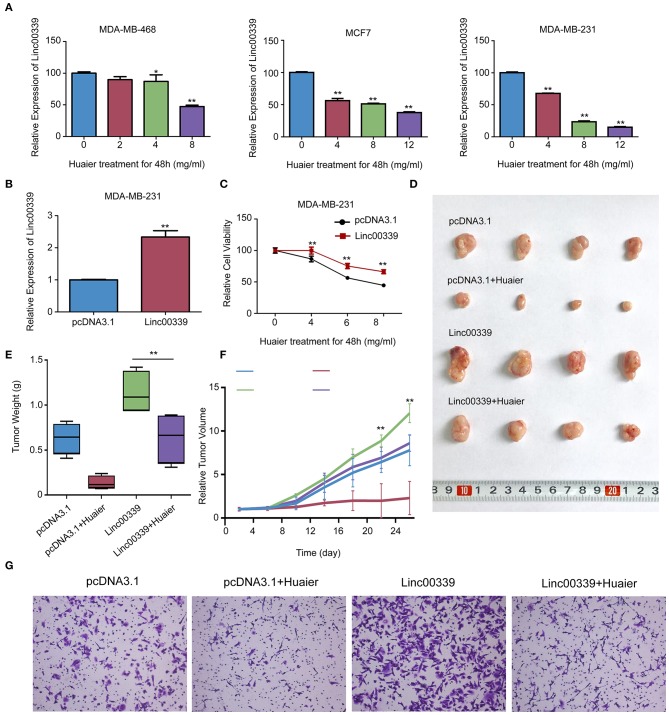
Linc00339 induced the chemoresistance of breast cancer cells to Huaier *in vivo*. **(A)** Huaier decreased the expression level of linc00339 in MDA-MB-468, MCF7 and MDA-MB-231. **(B)** Transfection efficacy of linc00339 overexpression in MDA-MB-231. **(C)** Overexpression of linc00339 inhibited the cytotoxicity of Huaier in MDA-MB-231. **(D)** *In vivo* assay indicated that linc00339 suppressed the effects of Huaier. **(E)** Tumor weights in the four groups. **(F)** Growth curves of tumor volumes in the four groups. **(G)** Transwell assay showed the migration of MDA-MB-231 cells. The experiments were performed in triplicate and data were presented as the mean ± SD of three separate experiments. ^*^p < 0.05; ^**^p < 0.01.

To validate the mechanism underlying the effect of Huaier, we first examined the levels of linc00339 in Huaier treated breast cancer cell lines. As shown in Figure 8A, Huaier could suppress the levels of linc00339 in MDA-MB-231, MDA-MB-468, and MCF7 in a dose-dependent manner. Overexpression of linc00339 could inhibit the cytotoxic effects of Huaier (Figures 8B,C). As shown in Figure 8D, linc00339 could improve the chemoresistance of breast cancer to Huaier *in vivo*. After overexpression of linc00339, the tumor weights of breast cancer xenografts were increased (Figure 8E). The same effects were also observed in the growth curves (Figure 8F). Furthermore, linc00339 could reverse the inhibitory effects of Huaier on cell migration (Figure 8G).

**Figure 9:**
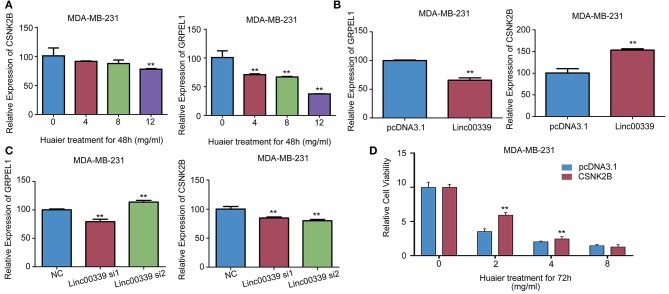
CSNK2B was the downstream target of linc00339. **(A)** The effects of Huaier on the expression levels of CSNK2B and GRPEL1 in MDA-MB-231. Overexpression **(B)** or suppression **(C)** of linc00339 on the expression levels of CSNK2B and GRPEL1 in MDA-MB-231. **(D)** Overexpression of CSNK2B rescued the effects of Huaier on cell proliferation. The experiments were performed in triplicate and data were presented as the mean ± SD of three separate experiments. ^**^p < 0.01.

CSNK2B and GRPEL1 were the potential targets of linc00339. As shown in Figure 9A, Huaier could decrease the expressions of CSNK2B and GRPEL1 in MDA-MB-231. Overexpression of linc00339 led to higher expression of CSNK2B, but lower level of GRPEL1 (Figure 9B). Inhibition of linc00339 could only decrease the level of CSNK2B, but not GRPEL1 (Figure 9C). Overexpression of CSNK2B could reverse the anti-proliferation effect of Huaier (Figure 9D). Therefore, CSNK2B, but not GRPEL1, was the exact downstream target of linc00339.

**Figure 10:**
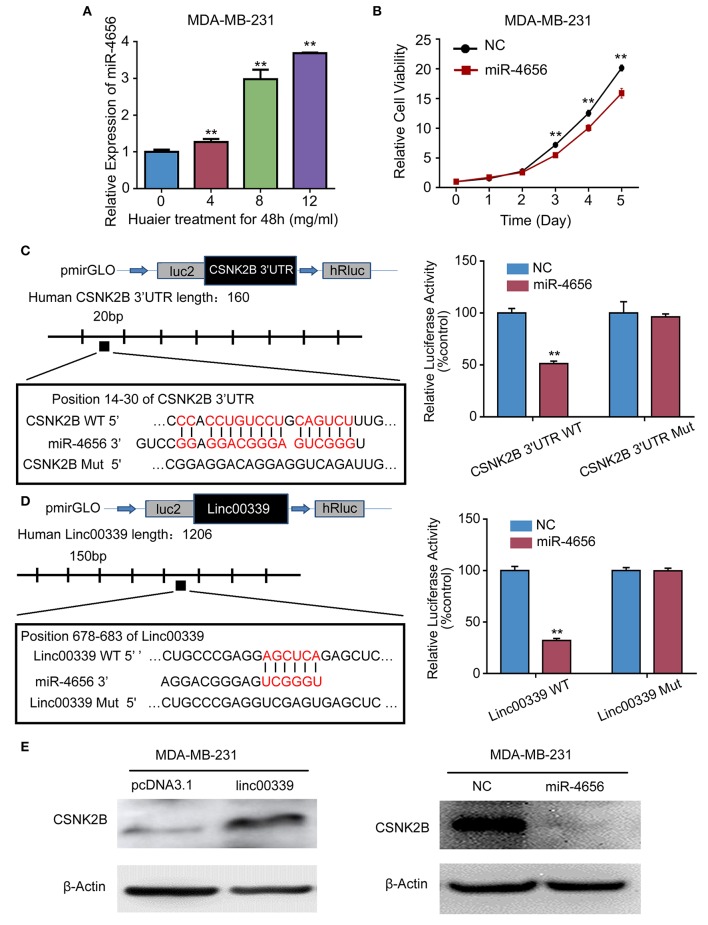
miR-4656 mediated the interaction between linc00339 and CSNK2B. **(A)** Huaier increased the level of miR-4656 in MDA-MB-231. **(B)** The effects of miR-4656 on cell proliferations of MDA-MB-231. **(C)** Predicted targeting sequence of miR-4656 at nucleotides 14–30 of the CSNK2B 3′UTR. Luciferase assay revealed that miR-4656 directly targeted CSNK2B 3'UTR. **(D)** Predicted targeting sequence of miR-4656 at nucleotides 678–683 of linc00339. Luciferase assay revealed that miR-4656 directly targeted linc00339. **(E)** The effect of linc00339 and miR-4656 on the expression of CSNK2B. Data represent means ± SD of at least three independent experiments. ^**^P < 0.01. NC, negative control. Wt: wild type; Mut: mutant type.

**Figure 11:**
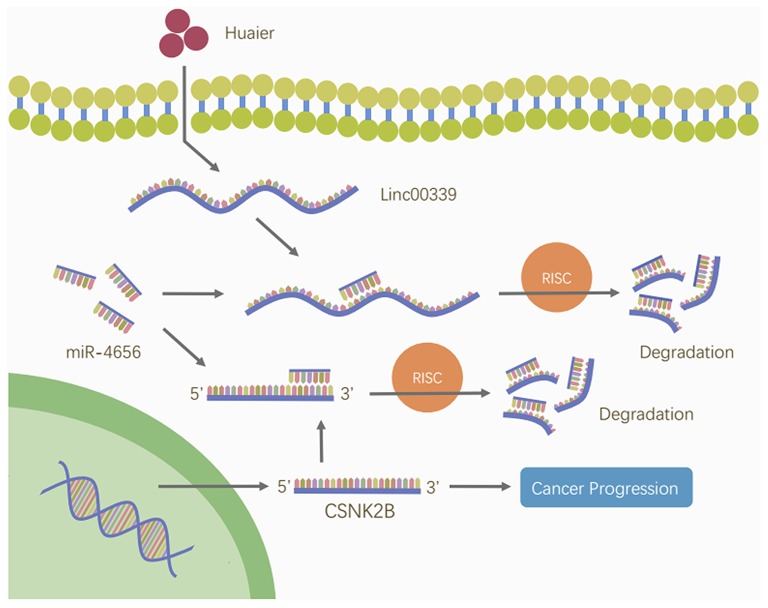
Huaier inhibited breast cancer progression through linc00339/miR-4656/CSNK2B signaling pathway. Huaier decreased the level of linc00339. Linc00339 sponged miR-4656, and enhanced the function of CSNK2B, thereby accelerating breast cancer progression.

To reveal the detailed mechanism underlying the regulation of linc00339 on CSNK2B, we performed *in silico* analysis. And miR-4656 was identified. We assumed that linc00339 could function as a ceRNA of miR-4656 and promote the function of CSNK2B. As shown in Figure 10A, Huaier could increase the level of miR-4656 in a dose-dependent manner. Overexpression of miR-4656 promoted the proliferation of breast cancer cells (Figure 10B). To confirm the direct effects of miR-4656 on CSNK2B, we cloned the predicted binding sites of miR-4656 into the pmirGLO vector. As shown in Figure 10C, cells co-transfected with miR-4656 and CSNK2B 3′UTR WT vector gained a significantly lower luciferase activity than those transfected with CSNK2B 3′UTR Mut vector. Same result was also observed in the binding between miR-4656 and linc00339 (Figure 10D). Additionally, overexpression of linc00339 could increase the levels of CSNK2B, and miR-4656 inhibited the expression of CSNK2B (Figure 10E). In conclusion, linc00339/miR-4656/CSNK2B signaling pathway played a critical role in the effect of Huaier (Figure 11).

## Discussion

Recently, the anti-cancer effects of Huaier have attracted increasing attentions (34). However, the molecular mechanisms underlying the functions of Huaier have not been extensively studied. Previously, through using microarray profiling, we identified the multi-target effects of Huaier (17). However, the roles of non-coding RNAs in Huaier-induced cell death have not been well-studied. To comprehensively understand the molecular pathways induced by Huaier, we performed microarray profiling including the data from lncRNA, miRNA, and mRNA. We identified several hub genes involved in the effects of Huaier, and used bioinformatic analysis to annotate their functions. To confirm our analysis, we performed *in vitro* and *in vivo* experiments to verify the role of linc00339/miR-4656/CSNK2B axis in the inhibitory effects of Huaier. Overall, our study established a molecular network affected by Huaier treatment, and uncovered a novel signaling pathway.

We explored the expression patterns of lncRNAs, miRNAs, and mRNAs between control group and Huaier treated group. Data from microarray profiles identified vast differentially expressed lncRNAs, miRNAs, and mRNAs. To handle the mass data, we first used GO and KEGG pathway analyses to divide the deregulated transcripts into various modules, suggesting that the effects of Huaier were involved in multiple aspects. Bioinformatic analyses, including WGCNA, were applied to build the coexpression networks and identify hub genes. Meanwhile, several subnetworks were constructed according to the k-score, and linc00339 was found to play a crucial role in the effect of Huaier. Then, to validate the accuracy of our analysis, we performed *in silico, in vitro*, and *in vivo* experiments to detect the effects of linc00339 on Huaier-induced cytotoxicity and explore the downstream targets. Public databases were applied to evaluate the clinical significance of the potential targets. Finally, linc00339/miR-4656/CSNK2B signaling pathway was identified and proved to mediate the inhibitory function of Huaier on breast cancer cells.

Emerging evidence indicated the important roles of non-coding RNAs in cancer development and progression (35, 36). Non-coding RNAs could be divided into two groups based on the number of nucleotides, including the miRNA group (nearly 22 bp) and the lncRNA group (>200 bp). Previous studies demonstrated that non-coding RNAs could act as breast cancer biomarkers and regulate chemoresistance in breast cancer cells (37, 38). We reported that lncRNA SNHG16 was upregulated in breast cancer tissues compared with the paired non-cancerous tissues (39). Overexpression of SNHG16 could promote the migration of breast cancer cells through modulating miR-98/E2F5 axis (39). Additionally, Huaier could inhibit the proliferation of breast cancer cell through regulating lncRNA-H19/miR675-5p pathway (22). Therefore, we supposed that non-coding RNAs played a critical role in Huaier-induced anti-tumor effect. In the present study, we revealed the molecular mechanisms of Huaier, through lncRNAs, miRNAs, and mRNAs. By extensively analyzing the coexpression network, linc00339 was identified as the core effector. As shown in our data, overexpression of linc00339 could reduce the inhibitory effect of Huaier. Inhibition of linc00339 promoted the chemosensitivity of breast cancer cells to Huaier. These data confirmed the rationality of our bioinformatic analysis.

LncRNA could regulate the progression of cancer through diverse mechanisms (40, 41). An important mechanism of lncRNAs was their activity as miRNA sponges (42, 43). Linc00339, also known as HSPC157, was located at 1p36. Previous studies showed that linc00339 was involved in the development of endometriosis and progression of cancers (44, 45). According to our study, linc00339 was increased in breast cancer cell lines compared with the normal epithelial cell, and linc00339 could promote triple-negative breast cancer progression through regulating miR-377-3p (46). In the present study, we further examined the mechanisms underlying the effects of linc00339 on Huaier-induced cytotoxicity. From the coexpression molecular network, multiple mRNAs were shown to be correlated with linc00339. According to the weight, we evaluated the impacts of candidate mRNAs on clinical prognosis. CSNK2B and GRPEL1 were identified. Overexpression of linc00339 induced the increased level of CSNK2B and decreased level of GRPEL1. Through searching the public database and luciferase assay, the linc00339/miR-4656/CSNK2B signaling pathway was found and gain-of-function experiments demonstrated that linc00339/miR-4656/CSNK2B axis played a critical role in regulating the function of Huaier.

In the present study, we used bioinformatic analysis and identified the core signaling pathway mediated the function of Huaier. Through using *in vitro* and *in vivo* experiments, the biological function of linc00339/miR-4656/CSNK2B axis was confirmed. Therefore, our study provided a reference for the analysis of high-throughput screening data and a novel signaling pathway in the treatment of breast cancer. Further investigations were needed to identify the responsible ingredient in Huaier and explore the possible molecular mechanisms.

## Data Availability Statement

All data generated or analyzed during this study were included in this published article.

## Ethics Statement

The animal study was reviewed and approved by the Ethics Committee on Scientific Research of Shandong University, Qilu Hospital.

## Author Contributions

QY and WW designed the experiments. WW, XW, CL, and TC performed the experiments and analyzed the data. WW, XW, and NZ prepared the manuscript draft. YLiang, YLi, XS, HZ, and YLiu set up the experiments and repeated the key experiments. QY, WW, XW, WZ, BC, and LW conceived the work, analyzed the data, and prepared the manuscript. All authors critically revised the manuscript, approved the final version, and agreed to be accountable for all aspects of the manuscript.

### Conflict of Interest

The authors declare that the research was conducted in the absence of any commercial or financial relationships that could be construed as a potential conflict of interest.
